# Reducing mother to child HIV transmission: a successful experience

**DOI:** 10.1186/1471-2334-14-S2-P35

**Published:** 2014-05-23

**Authors:** A Ouyahia, M Rais, A Gasmi, S Mechakra, A Lacheheb

**Affiliations:** 1 Ferhat Abbes University, Medicine Faculty, Setif, Algeria

## Background

Mother-to-child transmission of HIV remains the main source of pediatric HIV infection, women constituted 50% of infected people in Algeria, mostly in the reproductive age group.

## Objectives

Mandatory HIV screening in pregnancy, as part of mother-to-child transmission (MTCT) prevention, is useful to increase the number of patients under follow-up. Our work aimed to describe MTCT prevention interventions and outcome of newborns during 24-months follow-up.

## Methods

From 99 HIV women followed between 2002 and 2013 to the HIV unit of the teaching hospital of Setif, 10 pregnant women were identified. Pregnancies and deliveries (over 15 days) that occurred before referral to our institution were excluded from the study.

We used data collected in the patients' medical files, Epi info 3.5 version was used for statistical analysis.

## Results

10 mothers had 14 pregnancies, resulting in a total of 14 live born children.

The median age was 19.5 years, 7 were married and 3 were single. The most frequent transmission route was heterosexual (90 %). 3/10 fathers were unaware of HIV maternal status.

70 % had been previously diagnosed with HIV. All had HAART as MTCT prevention, with AZT+3TC+LPV/r; median viral load (VL) at delivery was below 1000 cp/mL in 100% of patients. Cesaerean-section was performed in 28.5 % of deliveries.

3 women were diagnosed after vaginal delivery, PMTCT started in post partum, median viral load after delivery was 7800 cp /mL. All neonates benefited of post natal antiretroviral prophylaxis (AZT during 6 weeks) and fed with formula milk. Mean newborn weight was 3030 g and HIV-PCR at birth, 3 months, 6 months of age was negative in 13 infants. All mother-infant pairs maintained follow-up at 24months except for one child who died at 6 months age.

## Conclusion

This important reduction in MTCT is a result of antenatal diagnosis and provision of antiretroviral prophylaxis and treatment for mothers and infants. It highlights the necessity of revisiting the debate of voluntary versus mandatory HIV/AIDS testing during pregnancy.

